# Diagnostic utility of transit time flow measurement for predicting early coronary bypass graft occlusion

**DOI:** 10.1016/j.xjon.2026.101955

**Published:** 2026-07-08

**Authors:** Rosa Kolb, Islam Salikhanov, Brian M. Mawad, Luise Voehringer, Ion Vasiloi, Saskia Rabe, Brigitta Gahl, Christian Mueller, Denis Berdajs

**Affiliations:** a CABG&Trop Research Group Department of Cardiac Surgery, University Hospital Basel, Basel, Switzerland; b Surgical Outcome Research Center Basel, University Hospital Basel, Basel, Switzerland; c Cardiovascular Research Institute Basel and Department of Cardiology, University Hospital Basel, Basel, Switzerland

**Keywords:** coronary artery bypass grafting, graft occlusion, hemodynamics, pulsatility index, transit-time flow measurement

## Abstract

**Objective:**

The study objective was to evaluate the predictive value of intraoperative graft flow and pulsatility index for early in-hospital graft occlusion.

**Methods:**

We prospectively included 634 patients undergoing isolated coronary bypass grafting surgery between 2020 and 2025. To assess bypass flow, intraoperative transit-time flow measurements were performed. Graft patency was determined in all patients by coronary computed tomography angiography or coronary angiography at discharge. Receiver operating characteristic analysis using the Youden index identified optimal intraoperative flow and pulsatility index thresholds for predicting in-hospital graft occlusion. A logistic regression-based nomogram using flow and pulsatility index was constructed to estimate occlusion risk, and multilevel mixed-effects models were applied to assess independent predictors of graft failure.

**Results:**

The incidence of early graft occlusion was 7.9% (n = 50) and associated with female sex, hypercholesterolemia, longer surgery, reoperations, and prolonged hospital stay. Venous grafts were associated with higher occlusion rates at the patient level (6.5% vs 3.0%, *P* = .004), with a similar trend observed at the graft level (3.1% vs 1.8%, *P* = .07). In conduit-specific analysis, every 10 mL/min increase in venous graft flow was associated with a 38% reduction in occlusion (odds ratio, 0.62; *P* < .001), whereas arterial grafts showed no such association with intraoperative flow or the pulsatility index. Optimal prognostic thresholds for venous grafts were identified at a mean flow less than 50 mL/min and a pulsatility index greater than 1.7.

**Conclusions:**

The incidence of early graft occlusion is significantly higher in venous conduits. Occlusion is associated with intraoperative graft flow, whereas no statistically significant relationship with these parameters was detectable in arterial grafts in this cohort.

**Figure:**
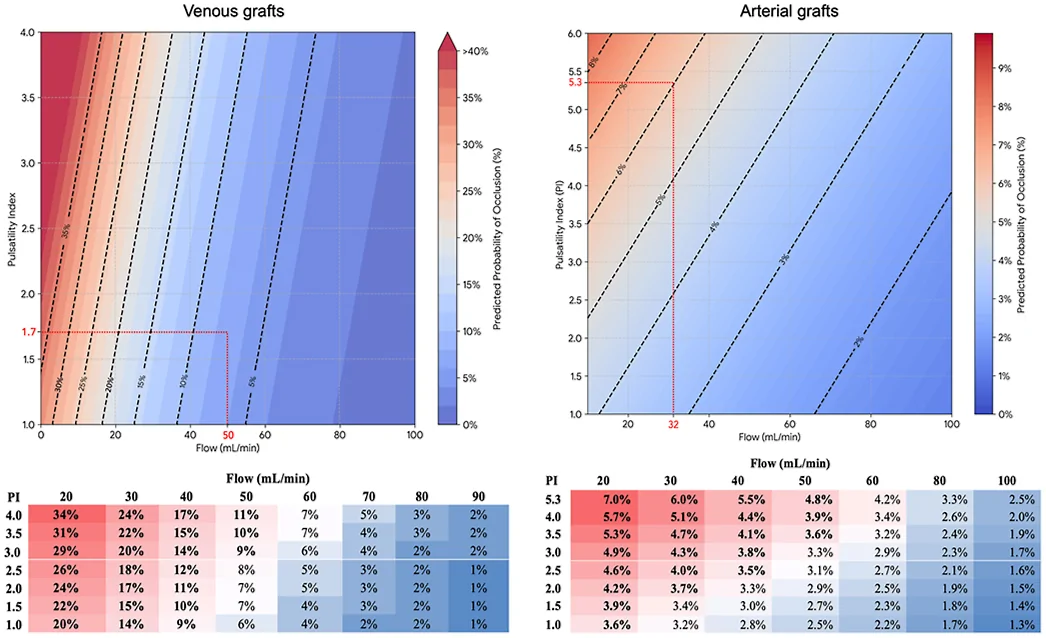
Conduit-specific nomograms for predicting early bypass graft occlusion risk.

**Central Message**

Intraoperative graft flow predicts early venous but not arterial bypass graft occlusion, mandating conduit-specific hemodynamic thresholds for quality control in coronary surgery.

**Perspective**

Current guidelines recommend uniform transit-time flow thresholds for all bypass grafts, yet venous and arterial conduits exhibit fundamentally distinct hemodynamic behavior. This study identifies conduit-specific thresholds that perform best in venous grafts and do not discriminate in arterial grafts within the observed range, supporting stratified rather than uniform intraoperative benchmarks for early graft failure.

Early silent graft occlusion after coronary artery bypass grafting (CABG) remains a critical complication, compromising procedural success and increasing the risk of prolonged hospitalization, recurrent angina, reintervention, and major adverse cardiac and cerebrovascular events.^1^, ^2^, ^3^ Pilot studies have suggested that even with modern surgical techniques, the incidence of early bypass graft occlusion may be as high as 5 to 10%.^1^, ^2^, ^3^ Two physiological intraoperative measurements are routinely obtained to noninvasively assess bypass graft function: bypass blood flow and pulsatility index (PI), with lower flow and higher PI found to be associated with a higher risk of graft occlusion.^4^^,^^5^ Current European Society of Cardiology/European Association for Cardio-Thoracic Surgery Guidelines on myocardial revascularization provide a Class IIa recommendation for transit-time flow measurement (TTFM) for intraoperative quality control in CABG.^6^ Although guidelines recommend the measurement, interpretation relies on a uniform set of values (flow <20 mL/min, PI >5.0) applied indiscriminately to physiologically distinct venous and arterial conduits.^7^ Attempts to define stratified thresholds, such as less than 11.5 mL/min for arteries versus less than 26 to 36 mL/min for veins, are scarce and controversial, and their reliability is further limited by imperfect verification of early bypass occlusion, because symptom-driven surveillance fails to capture early asymptomatic occlusions.^3^^,^^4^^,^^8^, ^9^, ^10^ Therefore, the aim of our study is to address these scientific and medical gaps by integrating routine TTFM with early systematic postoperative coronary imaging for all patients, capturing the true incidence of early graft failure and defining hemodynamic criteria for venous and arterial grafts.

## Materials and Methods

### Study Design and Population

We prospectively enrolled consecutive adult patients undergoing isolated CABG between April 2020 and August 2025 who had postoperative coronary computed tomography angiography (cCTA) or coronary angiography available to assess in-hospital graft occlusion. Per institutional protocol, all isolated CABG patients undergo 256-slice cCTA before discharge, interpreted by dedicated cardiologists and radiologists. The study received ethics approval from the Ethics Committee of Northwest and Central Switzerland under Project ID 2023-01850. Written informed consent was obtained from all participants.

### Graft Patency Assessment

Single, sequential, and composite (T- and Y-graft) configurations were included; each distal anastomosis and each limb of a composite graft was evaluated individually on predischarge cCTA, and the conduit was classified as occluded if any segment of the bypass body or any distal anastomosis showed absent contrast opacification. Intraoperative TTFM was recorded at the proximal hood of the conduit, reflecting cumulative outflow across all distal limbs. In our study, perioperative myocardial infarction was defined per the Fourth Universal Definition as postprocedural troponin elevation more than 10 times the upper reference limit in patients with normal baseline, plus new ischemia, imaging abnormalities, or angiographic complications.^11^

### Intraoperative Hemodynamic Measurements

Intraoperative graft flow and PI were measured by TTFM immediately after weaning from cardiopulmonary bypass and completion of protamine reversal, once stable hemodynamics were achieved (target heart rate ∼90 beats/min and arterial blood pressure ∼120/80 mm Hg). All intraoperative TTFM measurements were performed with the Medistim system (Medistim ASA), in routine institutional use since 2010, with 3.5-mm and 2-mm probes selected to match conduit caliber. Conduit-specific measurement of flow was recorded in mL/min, and PI was measured as (peak systolic flow – end-diastolic flow)/mean flow.

### End Points

The primary end point was early in-hospital graft occlusion, defined as complete absence of contrast opacification within the bypass graft or distal runoff on postoperative imaging. Secondary end points included conduit-specific differences in TTFM parameters for predicting early graft failure.

### Statistical Analysis

Continuous variables are reported as mean (SD) or median (IQR) and categorical variables as percentages. Continuous variables were compared using the Student *t* test or Mann–Whitney *U* test depending on the normality of distribution, and categorical data were analyzed using the chi-square test or Fisher exact test for low expected frequencies. Multivariable logistic regression analysis was performed to assess independent predictors of graft occlusion, including both intraoperative mean graft flow and PI as continuous variables. To account for clustering of grafts within patients, multilevel mixed-effects logistic regression models (with patient as random intercept) were used. These models specifically tested interactions between conduit type and hemodynamics (flow/PI), and between surgery duration and recent myocardial infarction. Diagnostic utility was evaluated using receiver operating characteristic analysis (area under the curve [AUC]) and the Youden Index to identify optimal cutoffs. Clinical risk nomograms were generated based on predicted probabilities from these models. Analyses were performed using Stata 18.0.

## Results

### Study Cohort and Clinical Outcomes

During the study period, 695 patients underwent isolated CABG. We excluded 61 patients (8.8%) who did not receive postoperative patency assessment due to either a known contrast allergy or severely impaired renal function (defined as an estimated glomerular filtrate rate <30 mL/min/1.73 m^2^). The study cohort consisted of 634 patients (2116 total grafts) treated at the University Hospital Basel from April 2020 to August 2025. The mean age was 67 (9.2) years, and 16% were female (Table 1). Although early in-hospital graft occlusion was identified in 50 patients (7.9%), the vast majority were asymptomatic; only 6 patients developed symptoms prompting urgent angiography, resulting in percutaneous coronary intervention (n = 3) or redo CABG (n = 3). Baseline characteristics were well balanced between groups, except for female sex (28% vs 15%; *P* = .03) and hypercholesterolemia (84% vs 69%; *P* = .04), which were significantly more prevalent in the occlusion group. At the patient level, venous graft occlusion occurred in 6.5% of patients (31/474), whereas arterial graft occlusion was observed in 3.0% of patients (19/634), corresponding to more than a 2-fold higher risk for venous graft occlusion (odds ratio [OR], 2.33; 95% CI, 1.25-4.39; *P* = .004). When analyzed at the level of individual conduits, the graft-level analysis showed a higher occlusion rate in venous conduits (33/1076, 3.1%) compared with arterial grafts (19/1040, 1.8%), representing 1.7-fold increased odds of failure (OR, 1.70; 95% CI, 0.93-3.19; *P* = .07). Intraoperatively, patients in the occlusion group required significantly longer surgical times (248 [51] vs 230 [56] minutes; *P* = .03) and received a higher mean number of distal anastomoses (3.24 [1.01] vs 2.88 [0.94]; *P* = .001), whereas the use of the left internal thoracic artery was significantly less frequent in the occlusion group (92% vs 98%, *P* = .04) (Table 2). Logistic regression confirmed that the number of distal anastomoses was a significant risk factor: For every additional distal anastomosis performed, the odds of early graft occlusion increased by 48% (OR, 1.48; 95% CI, 1.09-2.01; *P* = .01). In-hospital major adverse cardiac and cerebrovascular events, including stroke and myocardial infarction, were similar, showing no differences between groups.

**Table 1:** Preoperative patient characteristics

Variable	Patients with patent grafts (N = 584)	Patients with occluded grafts (N = 50)	*P* value
Age, y	67 (9.3)	66 (8.9)	.26
Female	87 (15%)	14 (28%)	**.03**
BMI	27 (4.3)	27 (3.4)	.33
Diabetes	232 (40%)	19 (38%)	.88
Current smoker	152 (26%)	12 (24%)	.50
Hypertension	454 (78%)	37 (74%)	.60
Hypercholesterolemia	404 (69%)	42 (84%)	**.04**
Creatinine clearance (mL/min)	91 (32)	89 (26)	.74
Chronic lung disease	42 (7.2%)	3 (6.0%)	>.99
Angina (CCS 3-4)	268 (46%)	23 (46%)	.59
NYHA 3-4	78 (13.3%)	4 (8.0%)	.61
Previous MI	297 (51%)	25 (50%)	>.99
Previous PCI	79 (14%)	8 (16%)	.67
Extracardiac arteriopathy	135 (23%)	11 (22%)	>.99
Previous cerebrovascular event	42 (7.2%)	4 (8.0%)	.78
No LMS disease	80 (14%)	8 (16%)	.67
Ejection fraction value (%)	54 (11)	54 (11)	.83

Comparison of baseline characteristics between patients with and without early graft occlusion. Data are presented as n (%) for categorical variables (representing column percentages), mean (SD) for normally distributed continuous variables, and median (IQR) for non-normally distributed continuous variables. Bolded values indicate statistical significant differences. *BMI*, Body mass index; *CCS*, Canadian Cardiovascular Society; *NYHA*, New York Heart Association; *MI*, myocardial infarction; *PCI*, percutaneous coronary intervention; *LMS*, left main stem.

**Table 2:** Intraoperative patient characteristics

Variable	Patients with patent grafts (N = 584)	Patients with occluded grafts (N = 50)	*P* value
Duration of surgery (min)	230 (56)	248 (51)	**.03**
Operation urgency			.90
Elective	390 (67%)	33 (66%)	
Urgent	146 (25%)	14 (28%)	
Emergency	46 (7.9%)	3 (6.0%)	
Salvage	2 (0.34%)	0 (0.00%)	
Perfusion			.95
Off-pump	150 (26%)	12 (24%)	
On-pump	434 (73%)	38 (76%)	
CPB time (min)	105 (30)	111 (31)	.19
Duration of aortic crossclamp (min)	69 (22)	74 (19)	.22
Surgical access			.80
Full median sternotomy	542 (93%)	48 (96%)	
Minithoracotomy	40 (6.8%)	2 (4.0%)	
Conduit use (patient-level)			
Right ITA	132 (23%)	15 (30%)	.23
Left ITA	571 (98%)	46 (92%)	**.04**
Radial	68 (12%)	5 (10%)	>.99
Long/great SV	431 (74%)	43 (76%)	.06
Number of distal anastomoses	2.88 (0.94)	3.24 (1.01)	**.01**
EuroSCORE II	1.5 (0.88-2.6)	1.4 (0.92-2.5)	.56

Comparison of intraoperative variables between patients with and without early graft occlusion. Data are presented as n (%) for categorical variables (representing column percentages), mean (SD) for normally distributed continuous variables, and median (IQR) for non-normally distributed continuous variables. Bolded values indicate statistical significant differences. *CPB*, Cardiopulmonary bypass; *ITA*, internal thoracic artery; *SV*, saphenous vein; *EuroSCORE II*, European System for Cardiac Operative Risk Evaluation II.

Postoperatively, graft-related reinterventions occurred in 10% of patients in the occlusion group, compared with 0.17% in the open-graft group (*P* < .001). There were significantly elevated biomarkers of myocardial injury, including peak creatine kinase–MB isoenzyme (109 vs 31 units; *P* < .001) and high-sensitivity troponin T (931 vs 529 units; *P* = .004). The median hospital length of stay was significantly prolonged in the occlusion group (9.0 vs 7.0 days; *P* < .001) (Table 3).

**Table 3:** Postoperative patient characteristics

Variable	Patients with patent grafts (N = 584)	Patients with occluded grafts (N = 50)	*P* value
New permanent pacemaker	5 (0.86%)	0 (0.00%)	>.99
Atrial fibrillation	103 (18%)	10 (20%)	.70
ICD	5 (0.86%)	0 (0.00%)	>.99
Reinterventions for graft problems	1 (0.17%)	5 (10%)	**<.001**
Pericardial effusion/tamponade	16 (2.7%)	0 (0.00%)	.63
Maximum CK	793 (1062)	808 (466)	.93
Day of peak	1.3 (0.62)	1.2 (0.42)	.22
Maximum CK-MB, U/L	31 (49)	109 (109)	**<.001**
Maximum troponin T, ng/L	529 (885)	931 (1290)	**.004**
Pulmonary complication	28 (4.8%)	5 (10%)	.17
Mortality	0 (0.00%)	0 (0.00%)	>.99
Cerebrovascular event			.66
No	572 (98%)	49 (98%)	
TIA	6 (1.0%)	0 (0.00%)	
Stroke	6 (1.0%)	1 (2.0%)	
Length of stay (d)	7.0 (7.0-9.0)	9.0 (8.0-11)	**<.001**

Comparison of postoperative outcomes between patients with and without early graft occlusion. Data are presented as n (%) for categorical variables (representing column percentages), mean (SD) for normally distributed continuous variables, and median (IQR) for non-normally distributed continuous variables. Bolded values indicate statistical significant differences. *ICD*, Implantable cardioverter-defibrillator; *CK*, creatine kinase; *CK-MB*, creatine kinase–MB isoenzyme; *TIA*, transient ischemic attack.

### Bypass Hemodynamics

When analyzing all conduits collectively, median graft flow did not significantly differ between patent and occluded grafts (51 vs 53 mL/min; *P* = .33). Intraoperative TTFM revealed significant hemodynamic disparities in vein grafts, where occluded vein grafts exhibited significantly lower median flow (35 vs 62 mL/min; *P* < .001) and higher PI (1.9 vs 1.4; *P* = .01) compared with patent vein grafts. In contrast, all arterial conduits, the left internal thoracic artery, right internal thoracic artery, and radial artery, exhibited no significant hemodynamic discrimination between groups, displaying comparable median flow (44 vs 36 mL/min; *P* = .43) and PI (1.6 vs 1.5; *P* = .64) in patent versus occluded grafts. In conduit-stratified models, the protective benefit of higher graft flow was exclusive to venous conduits, where every 10 mL/min increase reduced occlusion risk by 38% (OR, 0.62; 95% CI, 0.50-0.76; *P* < .001), whereas the hemodynamic resilience of arterial grafts masked this protective association in the combined analysis. In mixed-effects models, the predicted probability of occlusion in venous grafts demonstrated a steep inverse relationship with flow, decreasing from 14% at 20 mL/min to 4% at 200 mL/min (Figure 1). In contrast, the risk for arterial grafts remained low and constant across the same flow range. Overall, the PI demonstrated lack of discriminative value, remaining constant at a median of 1.5 across both outcome groups (*P* = .81). However, venous grafts showed a significant risk escalation (9% to 17%) as PI increased from 0.5 to 9.5, in contrast to the hemodynamically independent risk profile observed in arterial conduits. As a secondary, exploratory observation, prolonged surgery duration was associated with a higher probability of occlusion, a risk gradient that was particularly pronounced in patients with recent myocardial infarction (Figure 2). This observation is presented as hypothesis generating and was not incorporated into the conduit-specific TTFM nomogram. A formal post hoc power calculation across the 1040 arterial grafts (19 events; event rate 1.8%; SD of arterial flow 26.5 mL/min; 2-sided α = 0.05; 80% power), based on the standard error of the logistic regression coefficient, showed that the arterial subgroup is powered to detect a minimum protective effect of OR 0.79 per 10 mL/min increase in flow (log OR, 0.24). On the log-odds scale that governs detectability in logistic regression, this minimum detectable effect is half the magnitude of the observed venous effect (OR, 0.62; log OR, 0.48), meaning the arterial sample could have detected only effects at least as strong as half the venous signal. Smaller arterial hemodynamic signals cannot be excluded, and the arterial finding is therefore framed as hypothesis generating rather than confirmatory.

**Figure 1:**
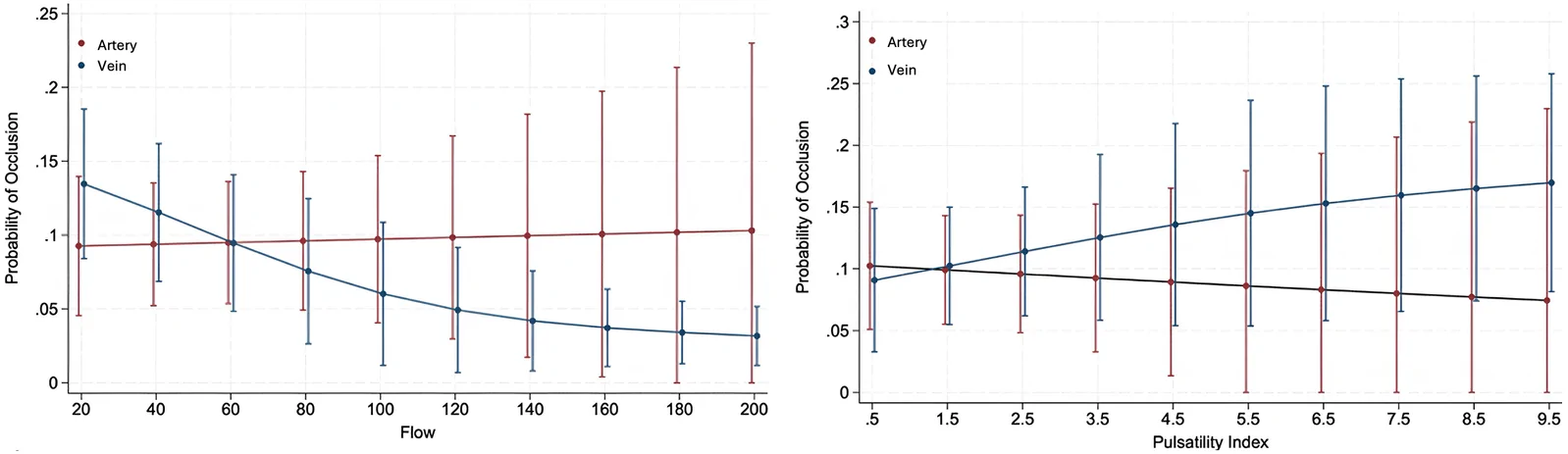
Predicted probabilities from mixed-effects logistic regression. Venous grafts (blue) show increased risk with lower flow and higher PI, whereas arterial grafts (red) remain hemodynamically stable. Error bars: 95% CI adjusted for clustering.

**Figure 2:**
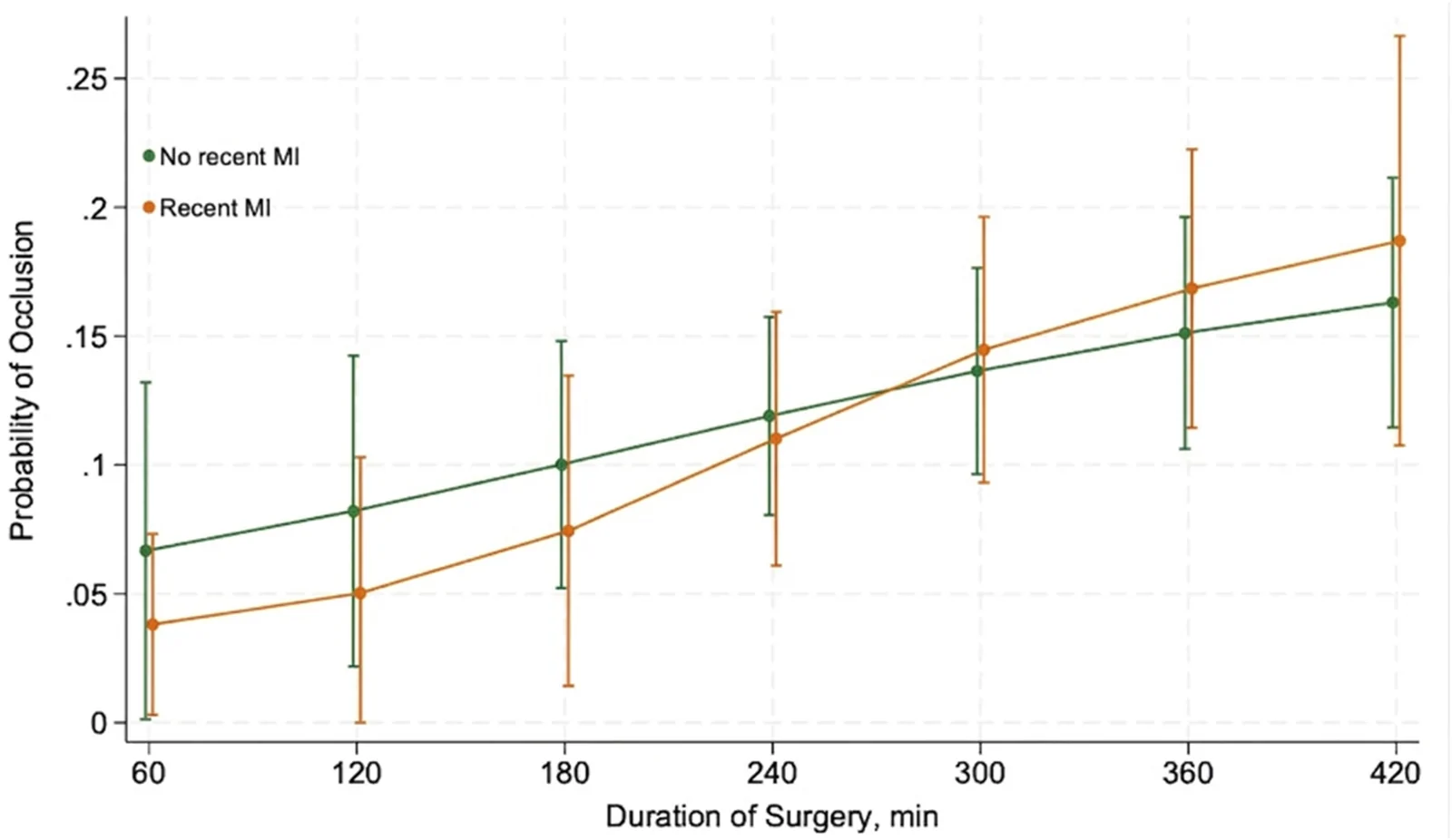
Predicted probabilities from mixed-effects logistic regression. Prolonged surgery increases occlusion risk, with a steeper gradient in patients with recent myocardial infarction (orange) compared with those without (green). Error bars: 95% CI adjusted for clustering.

### Nomogram for Estimation of Early Graft Occlusion

High and statistically significant predictive accuracy (AUC, 0.79; 95% CI, 0.72-0.87; *P* < .001) was achieved exclusively in the venous-specific analysis using Youden index–derived thresholds of mean flow less than 50 mL/min and PI more than 1.7 (predicted risk >8.5%) (Table 4). A paired DeLong test confirmed that the venous-specific multivariable model significantly outperformed the unified combined-conduit model in discriminating early venous graft occlusion (AUC, 0.79 vs 0.64; chi-square = 15.3; *P* < .001). The clinical risk nomogram is shown in Figure 3, where high-risk zones in venous grafts concentrate at flows less than 40 mL/min with PI greater than 2.0, whereas a definitive safe zone appears at flows more than 50 mL/min regardless of PI. In contrast, arterial grafts showed no such association with intraoperative flow or the PI, because neither standard regression models nor Youden index–derived thresholds (mean flow <32 mL/min, PI >5.3; predicted risk >3.5%) achieved statistical significance. Finally, combining arterial and venous grafts into a unified model further diminished predictive accuracy and failed to achieve statistical significance (Table 4).

**Table 4:** Comparative diagnostic accuracy of flow, pulsatility index, and combined risk models for venous and arterial grafts

Conduit type	Variable/model	Optimal cutoff	Sensitivity	Specificity	AUC (95% CI)	*P* value
Venous	Flow	<50 mL/min	77%	65%	0.71 (0.63-0.79)	<.01
	PI	>1.7	60%	68%	0.64 (0.55-0.73)	.24
	Multivariate risk model	>8.5%	73%	76%	0.79 (0.72-0.87)	<.001
Arterial	Flow	<32 mL/min	50%	72%	0.61 (0.49-0.73)	.24
	PI	>5.3	11%	100%	0.55 (0.45-0.65)	.40
	Multivariate risk model	>3.5%	39%	74%	0.54 (0.40-0.69)	.23
Combined	Flow	<32 mL/min	44%	84%	0.64 (0.60-0.68)	<.01
	PI	>2.4	26%	88%	0.57 (0.48-0.66)	.25
	Multivariate risk model	>11.0%	52%	77%	0.64 (0.50-0.78)	<.01

The venous multivariable model (incorporating mean flow and PI) demonstrated the highest diagnostic utility (AUC, 0.79, *P* < .001), whereas arterial parameters were nonsignificant. Multivariate risk model refers to the predicted probability of occlusion derived from logistic regression of both flow and PI, expressed as a percentage. *AUC*, Area under the curve; *PI*, pulsatility index.

**Figure 3:**
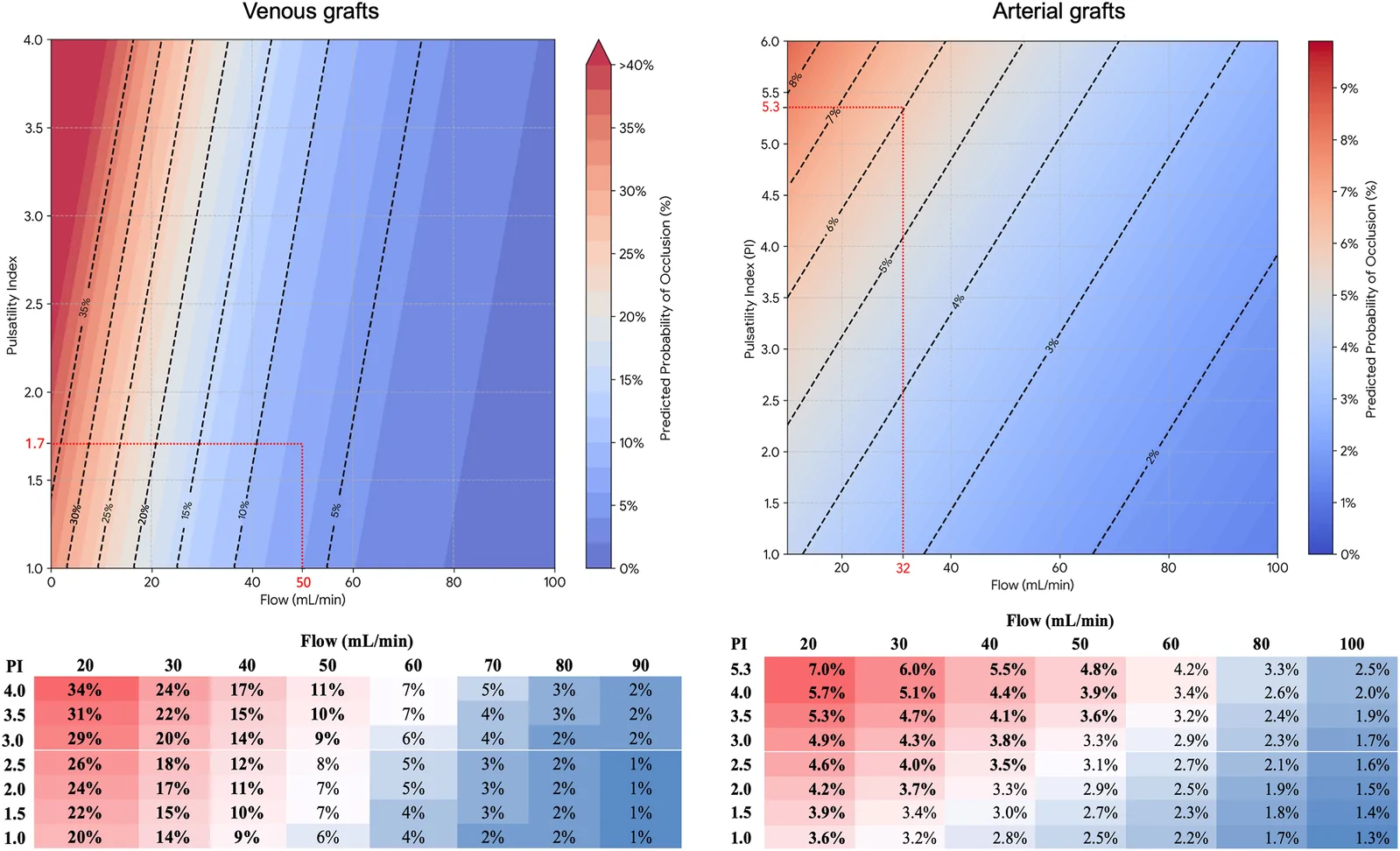
Predicted probabilities of graft failure based on mean flow and PI for (left) venous, and (right) arterial. Dashed contours and heat maps (red = higher risk) visualize the continuous risk gradient. Optimal thresholds are indicated: venous (flow < 50 mL/min, PI >1.7) and arterial (flow < 32 mL/min, PI >5.3).

## Discussion

This study provides a comprehensive analysis of in-hospital graft failure by correlating intraoperative hemodynamics with routine postoperative computed tomography imaging in 634 patients, enabling specific evaluation of 2116 total conduits (arterial and venous). We established a patient-level occlusion incidence of 7.9% and a graft-level incidence of 2.5%, confirming that early graft failure is a relevant, although often silent, complication. Venous conduits were significantly more vulnerable to early occlusion than arterial grafts (*P* < .05). Furthermore, our analysis demonstrated that graft failure is associated with patient characteristics, namely, a higher proportion of female patients and hypercholesterolemia, and procedural factors, including longer surgery duration and a higher number of anastomoses (*P* < .05) (Tables 1 and 2). Graft failure significantly compromised clinical recovery, resulting in increased reoperations, elevated cardiac enzymes, and prolonged length of stay (*P* < .05), underscoring the complex etiology of early graft failure. Consistent with prior studies, our data demonstrate a conduit-specific hemodynamic disparity, with venous grafts being the only conduits showing a significant reduction in flow in occluded versus patent grafts (35 vs 62 mL/min; *P* < .001). Confirming the protective value of higher hemodynamics, every 10 mL/min increase in venous graft flow was associated with a 38% reduction in occlusion (OR, 0.62; *P* < .001), whereas arterial grafts showed no such association with intraoperative flow or the PI. Occluded sequential vein grafts showed consistent patency of proximal and intermediate anastomoses with failure concentrated at the most distal anastomosis, supporting the principle that cumulative outflow preserves proximal sequential patency while the distal site remains vulnerable to local target-specific factors. Predischarge cCTA showed that failure occurred predominantly at the distal anastomosis or the distal segment of sequential grafts, with proximal aortic anastomoses uniformly patent; small-caliber distal targets and competitive native flow were the recurrent contributors. Our analyses indicate that determinants of occlusion are conduit-specific rather than territory-specific, because no regional effect on early graft failure was observed.^3^

Venous grafts showed strong predictability with cutoffs of flow less than 50 mL/min and PI more than 1.7 (AUC, 0.79), whereas arterial grafts exhibited lower thresholds (flow <32 mL/min, PI >5.3) and poor model performance likely due to the ability of arterial conduits to autoregulate caliber in response to competitive flow, unlike passive venous conduits. Merging these groups into a generic model compromised diagnostic accuracy (AUC, 0.64), reinforcing the necessity for stratification. The Youden index–derived optimal predicted-probability cutoff—defined as the threshold maximizing the sum of sensitivity and specificity—was more than 8.5% for venous graft occlusion in the mixed-effects model, compared with more than 3.5% for arterial grafts and more than 11.0% for the overall mixed model. This model underpins our nomogram, which visually integrates these parameters for rapid, point-of-care risk stratification of venous graft occlusion, particularly valuable given that early in-hospital failures are often asymptomatic (Figure 3).^4^

To bridge risk stratification and intraoperative action, we propose a provisional decision pathway that operationalizes the high-risk zone of the venous nomogram (flow <40 mL/min, PI >2.0) along 2 clinical axes assessed routinely at weaning from cardiopulmonary bypass: hemodynamic stability and the presence of ischemic signs on electrocardiogram or new wall-motion abnormalities on transesophageal echocardiography (Figure E1). In the hemodynamically stable patient without ischemic signs, intraoperative TTFM is repeated immediately before chest closure. In the hemodynamically stable patient with electrocardiogram changes or new wall-motion abnormalities, the graft is inspected for kinking or misalignment, and anastomotic revision is considered if no mechanical cause is identified. In the hemodynamically unstable patient without ischemic signs, TTFM is repeated after stabilization, and anastomotic revision is undertaken only if flow remains below 40 mL/min and PI above 2.0. In the hemodynamically unstable patient with electrocardiogram changes or new wall-motion abnormalities, anastomotic revision is performed directly.

Our results reinforce the pivotal role of TTFM in predicting early graft patency, aligning with reported occlusion rates of 5 to 10% driven by suboptimal hemodynamics.^9^^,^^10^^,^^12^^,^^13^ Although traditional guidelines lack conduit-specific standards, literature thresholds for failure vary significantly: Arterial risk cutoffs range from less than 11.5 to 26.5 mL/min, whereas saphenous veins require higher flows (∼31–36 mL/min) to maintain patency.^14^^,^^15^ Furthermore, recent studies challenge the classic PI less than 5.0 target, advocating for stricter thresholds near 2.9.^3^^,^^8^ Consistent with this, our cohort displayed a paradox where venous grafts exhibited higher median flows than arteries yet failed significantly more often, confirming that high intraoperative graft flow alone does not guarantee venous patency.^16^ Our identified venous thresholds (flow <50 mL/min, PI >1.7) are stricter than previously cited values, suggesting that veins require superior hemodynamics to compensate for their inherent biological vulnerability to shear stress and endothelial dysfunction.^13^^,^^17^ We attribute this divergence to 2 methodological differences. First, prior thresholds were derived in cohorts that performed symptom-driven, selective postoperative imaging, which misses asymptomatic early occlusions and may bias the failure-defining flow values downward; our routine predischarge cCTA in every patient captures silent failures and exposes the level of hemodynamic insufficiency that they require. Second, prior studies pooled arterial and venous conduits in a single threshold, diluting venous-specific signal because arterial grafts maintain low-flow patency through autoregulation; once stratified by conduit, venous thresholds rise as expected. The surgery duration by recent myocardial infarction interaction shown in Figure 2 is a secondary observation, which identifies a procedural-risk gradient that warrants dedicated follow-up in studies designed for perioperative myocardial infarction prevention.

### Limitations

This single-center, observational study is limited by the absence of external validation and the underrepresentation of female patients, which may influence generalizability. Furthermore, the low occlusion rate constrained statistical power for arterial analysis despite robust venous modeling. Although our combined model identified a generic 32 mL/min cutoff consistent with literature, this “one-size fits all” value proved to be a mathematical compromise: It reduced venous sensitivity from 77% to 44% and lacked relevance for resilient arteries, confirming the necessity of conduit-specific criteria.^12^^,^^18^ The conduit-specific thresholds and the venous nomogram are derived and internally validated within a single-institution dataset with features (universal predischarge cCTA, standardized TTFM measurement conditions, and 98% left internal thoracic artery use) that may not generalize to other centers. Prospective external validation in independent multi-institutional cohorts is essential before the proposed thresholds are integrated into clinical guidelines or routine intraoperative decision-making.^19^^,^^20^ Furthermore, patient-level intraoperative hemodynamic state and vasoactive support at the time of TTFM were not systematically captured and represent unmeasured confounders that should be addressed in future prospective work.^9^^,^^21^

## Conclusions

Applying uniform TTFM cutoffs oversimplifies risk assessment and compromises diagnostic precision. Early in-hospital graft occlusion is predominantly a venous phenomenon, driven by vein sensitivity to suboptimal hemodynamics in contrast to the resilient physiology of arterial conduits. The presented thresholds should be regarded as provisional and require prospective external validation before adoption into routine practice. These findings advocate for the adoption of conduit-specific benchmarks to optimize intraoperative quality control and reinforce the prioritization of arterial revascularization.

## Conflict of Interest Statement

The authors reported no conflicts of interest.

The *Journal* policy requires editors and reviewers to disclose conflicts of interest and to decline handling or reviewing manuscripts for which they may have a conflict of interest. The editors and reviewers of this article have no conflicts of interest.

## Figures and Tables

**Figure E1:**
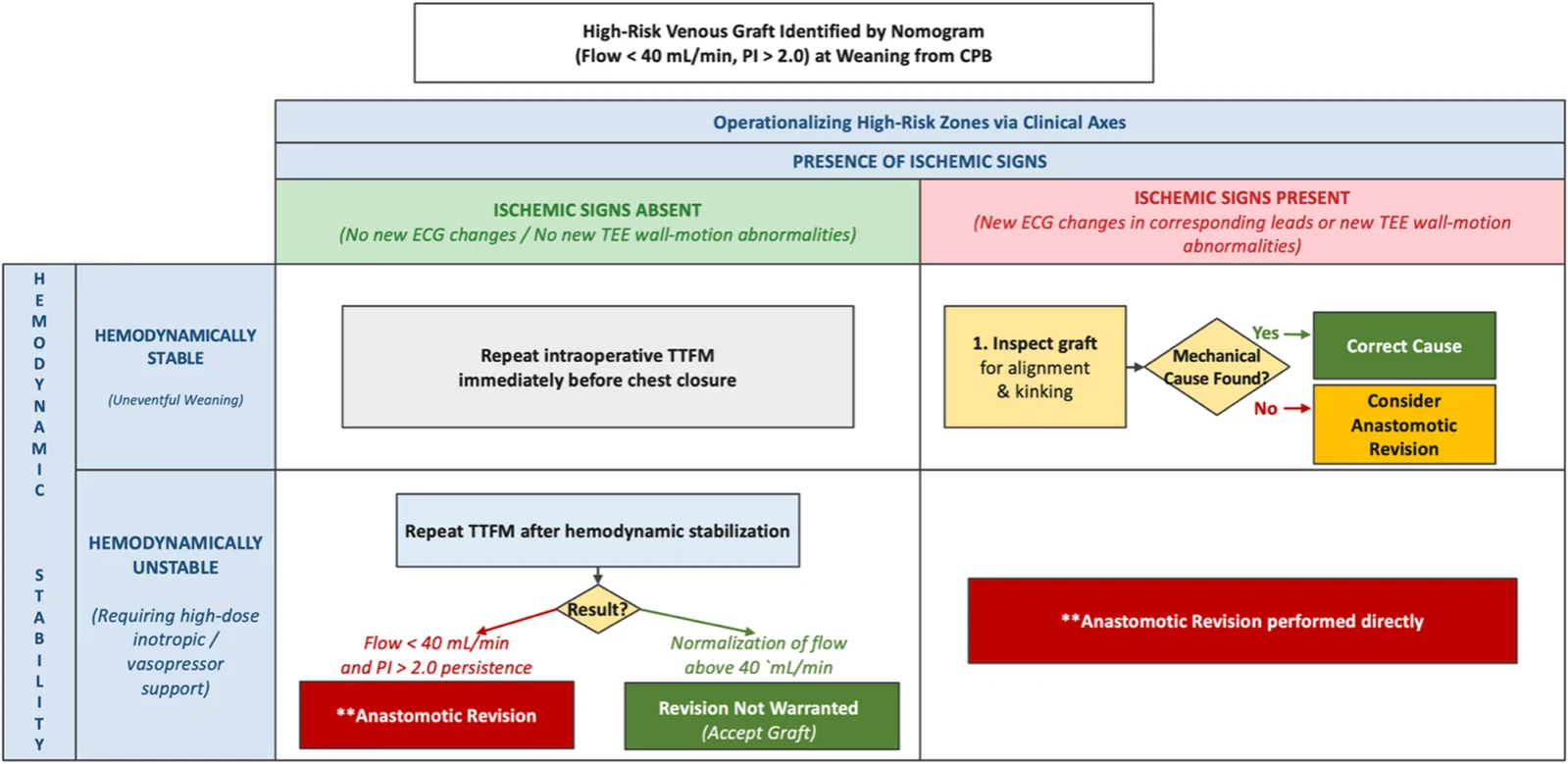
Provisional intraoperative decision pathway for high-risk venous grafts (flow <40 mL/min, PI >2.0). Algorithm for managing grafts with suboptimal TTFM parameters (mean flow <40 mL/min, PI >2.0) during cardiopulmonary bypass weaning. Actionable interventions are dictated by the presence of ischemia (electrocardiography/transesophageal echocardiogram) and hemodynamic stability. Immediate revision is required for overt ischemia or mechanical failure; unstable patients require hemodynamic optimization and repeat TTFM before final graft disposition. *PI*, Pulsatility index; *CPB*, cardiopulmonary bypass; *ECG*, electrocardiogram; *TEE*, transesophageal echocardiogram; *TTFM*, transit-time flow measurement.
